# Response surface methodology mediated optimization of Lignin peroxidase from *Bacillus mycoides* isolated from Simlipal Biosphere Reserve, Odisha, India

**DOI:** 10.1186/s43141-021-00284-2

**Published:** 2022-01-03

**Authors:** Subhashree Rath, Manish Paul, Hemanta Kumar Behera, Hrudayanath Thatoi

**Affiliations:** Department of Biotechnology, Maharaja Sriram Chandra Bhanja Deo University, Takatpur, Baripada, Odisha 757003 India

**Keywords:** Lignin, Lignin peroxidase, *Bacillus mycoides*, Dye decolourization, Enzyme activity, Response Surface methodology

## Abstract

**Background:**

Lignin is a complex polymer of phenyl propanoid units found in the vascular tissues of the plants as one of lignocellulose materials. Many bacteria secrete enzymes to lyse lignin, which can be essential to ease the production of bioethanol. Current research focused on the study of ligninolytic bacteria capable of producing lignin peroxidase (LiP) which can help in lignin biodegradation and bioethanol production. Ligninolytic bacterial strains were isolated and screened from the soil samples of Simlipal Biosphere Reserve (SBR), Odisha (India), for the determination of their LiP activity. Enzymatic assay and optimization for the LiP activity were performed with the most potent bacterial strain. The strain was identified by morphological, biochemical, and molecular methods.

**Results:**

In this study, a total of 16 bacteria (Simlipal ligninolytic bacteria [SLB] 1–16) were isolated from forest soils of SBR using minimal salt medium containing lignin. Out of the 16 isolates, 9 isolates showed decolourization of methylene blue dye on LB agar plates. The bacterial isolates such as SLB8, SLB9, and SLB10 were able to decolourize lignin with 15.51%, 16.80%, and 33.02%, respectively. Further enzyme assay was performed using H_2_O_2_ as substrate and methylene blue as an indicator for these three bacterial strains in lignin containing minimal salt medium where the isolate SLB10 showed the highest LiP activity (31.711 U/mg). The most potent strain, SLB10, was optimized for enhanced LiP enzyme activity using response surface methodology. In the optimized condition of pH 10.5, temperature 30 °C, H_2_O_2_ concentration 0.115 mM, and time 42 h, SLB10 showed a maximum LiP activity of 55.947 U/mg with an increase of 1.76 times from un-optimized condition. Further chemical optimization was performed, and maximum LiP activity as well as significant dye-decolourization efficiency of SLB10 has been found in bacterial growth medium supplemented individually with cellulose, yeast extract, and MnSO_4_. Most notably, yeast extract and MnSO_4_-supplemented bacterial culture medium were shown to have even higher percentage of dye decolourization compared to normal basal medium. The bacterial strain SLB10 was identified as *Bacillus mycoides* according to morphological, biochemical, and molecular (16S rRNA sequencing) characterization and phylogenetic tree analysis.

**Conclusion:**

Result from the present study revealed the potential of *Bacillus mycoides* bacterium isolated from the forest soil of SBR in producing LiP enzyme that can be evaluated further for application in lignin biodegradation and bioethanol production. Scaling up of LiP production from this potent bacterial strain could be useful in different industrial applications.

**Graphical Abstract:**

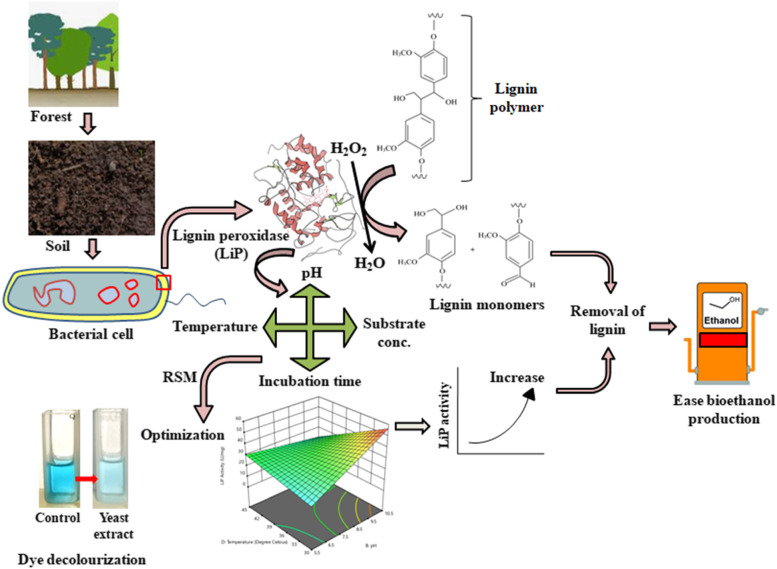

## Background

Fossil fuel serves as a major non-renewable source in global energy sector. It was a fundamental driver of the industrial revolution and the technological, social, and economic progress [1]. The excessive uses of this non-renewable fuel in industrial and transportation process results in environmental pollution through emitting greenhouse gases that are responsible for the climate change and global warming [2]. Apart from these, the fossil fuel reservoir is also depleting fast due to its over exploitation and necessitates search for an alternative source of renewable energy to overcome the energy crisis in future. In this context, lignocellulosic biofuel forms an alternative energy source which can substitute fossil fuel. Versatile ligninolytic microorganisms can generate renewable energy fuels form the lignocellulosic biomass (LCB) and biological wastes which can diminish the threatening concern of environmental pollution to a large extent [3]. Among others, the most favourable substitute for fossil fuel is bioethanol. Bioethanol, a form of biofuel produced from plant biomass sources, is ecofriendly as well as has the potential to simultaneously replace conventional fossil fuels and reduce the environmental concerns. In recent years, the production of biofuels using various microorganisms (both bacteria and fungus) has been steadily increasing. Due to metabolic diversity, different microorganisms are capable of producing bioethanol from various substrates such as sugary, starchy, and lignocellulosic biomasses. However, LCB serves as a potential source of bioethanol that is economic and non-competitive in comparison to food biomass [4].

LCB consist of cellulose, hemicellulose, and lignin, in which cellulose and hemicellulose are used for the production of bioethanol whereas lignin is considered as an impurity during bioethanol formation as it is a phenolic compound and contains no sugar in it [4–6]. Lignin present in the plant cell wall and one of the main components of all the vascular plant which serves as a structural constituent unit. This biopolymer acts as natural glue which holds cellulose and hemicellulose fibers together. It prevents easy accessibility of cellulase and hemicellulose enzymes towards their hydrolysis in the process of biofuel production. Therefore, lignin should be removed from cellulose and hemicellulose for maximum production of bioethanol. The separation of lignin from the LCB has several other advantages as it has many biotechnological and industrial applications. Chemical catalysts assisted lignin degradation, and thermochemical treatment requires severe chemical reaction conditions for highly efficient lignin depolymerization which causes damage to the environment. On the other hand, such adverse effect can overcome by using biological lignin depolymerization method that has now fascinated a growing consideration on lignin valorization [7].

The biological depolymerization process mainly involves the fungal-based degradation system and bacterial-based degradation system which are an efficient environment-friendly method for lignin valorization [7]. Therefore, research on the microorganism-based lignin degradation has received great attention [8]. Fungus and bacteria produce numbers of ligninase enzymes such as lignin peroxidase, manganese peroxidase, laccase, dye decolourizing peroxidase, and versatile peroxidase-significant enzyme for the degradation of lignin from LCB. Liginin peroxidase is one of the most well-known abundant and significant ligninase enzyme produced by many microorganisms including bacteria [5, 6]. Lignin peroxidase (LiP) (EC 1.11.1.14) enzyme belongs to the family of oxidoreductase that catalyzes the cleavage of different bonds present in lignin polymer using hydrogen peroxidase (H_2_O_2_) as oxygen acceptor [5]. The first LiP was discovered in white rot fungus *Phanerochaete chrysosporium* in its extra cellular medium [9]. Later isoforms of LiP are found in other fungi such as *Tinea versicolor*, *Phanerochaete sordida*, *Phlebia radiate*, *Phelibia tremellosa*, *Lentinula edodes*, *Sporotrichum pulverulentum*, *Pycnoporus cinnabarinus*, *Phelibia tremiellosus*, *Pleurotus* sp., and *Coriolus versicolor* [10, 11]. Apart from these fungi, the bacterial LiP enzymes involve in lignin degradation acquired great attention due to their heterologous expression compared with fungal ones [12]. There has been reported that the LiP enzymes mainly produced in Actinomycetes, α-proteobacteria, and γ-proteobacteria can degrade lignin efficiently [13].

The in vitro microbial LiP production mainly depends upon various physical and environmental parameters like carbon/nitrogen ratio, acidity or alkalinity of medium, presence of metal ions, temperature, incubation time, and oxygen content in the medium [12]. In the microbial LiP-mediated oxidative depolymerization process of lignin, LiP catalyzes the cleavage of different bonds present in lignin polymer using hydrogen peroxidase (H_2_O_2_) as an oxygen acceptor [5]. However, it has been reported that only some higher fungi and bacteria are effective in the degradation of lignin polymer through this oxidative reaction process. Although in-depth studies regarding this mechanism have been carried out repeatedly in the past 20 years, the mechanism has not yet been fully interpreted. To understand this brief mechanism of enzyme-mediated biodegradation of lignin, studies on the enzyme activity and its stimulatory factors should be undertaken. Optimization of growth factor to enable maximum production and activity of enzyme is mostly significant in this essence. Till date, most of the optimization studies for LiP have been reported only for fungal sources [14–18]. In case of ligninolytic bacteria, some reports are available on their isolation, characterization, and identification from different sources like decaying plant materials and soil samples [19–24]. However, the optimization of growth factors for the maximum lignin peroxidase activity from bacteria is infrequent and needs extensive studies.

Based on the current scenario, an attempt has been made to isolate ligninolytic bacteria from Similipal Biosphere Reserve (SBR), Odisha (India), and evaluate their LiP activity. The Similipal Biosphere Reserve located in the northern most part of Odisha extends over an area of 5578 km^2^ covering three districts, Mayurbhanj, Keonjhar, and Balasore. Its humid monsoon weather provides suitable conditions for the growth of numerous flora fauna and microorganisms [25]. The forest soils of SBR contain lignocellulosic leaf litter materials which provide a suitable habitat for the growth of ligninolytic microorganisms. Previous studies have reported the occurrence of cellulase- and xylanase-producing microorganisms from soils of SBR [26, 27]. It is apparent that lignin-degrading microorganism will be potentially available in forest soils contaminated with high content of decompose lignocellulosic materials. Based on the background, the present study is directed towards isolating, screening, and characterizing the most potent ligninolytic bacteria from SBR soil samples for evaluating lignin peroxidase activity which can be utilized for bioethanol and other value added product formation. Further, the optimization of LiP activity from the most efficient ligninolytic bacterium using response surface methodology was performed in a view to explore the use of isolated strain for the production of bioethanol from lignocellulosic waste.

## Methods

### Collection of soil sample

The soil samples underneath the plant mixed with scraps of decaying bark were freshly collected randomly under aseptic condition from 2 to 10 cm below the surface area. The soil samples were collected from seven different selected sites of Sitakunda and Lulung regions of SBR forest area situated in Mayurbhanj district of Odisha, India. SBR is a natural forest positioned at the latitude of 21° 10′ to 22° 12′ N and longitude of 85° 58′ to 86° 42′ E and expanded over a reserve forest area of 2750 km^2^ [24]. About 1 cm of the uppermost layer of the soil was taken out before collection of the sample. Each soil samples were put in a sterile zip locked polythene bags with proper labeling. These bags were packed in ice box and brought to the laboratory for the storage at 4 ± 0.1 °C for future analysis.

### Isolation of ligninolytic bacteria

#### Preparation of alkali lignin

The dried barks of tree *Peltophorum pterocarpum* (Radha chuda) were collected as a source of lignin. The barks were grinded to turn them into powder form followed by the acid pretreatment. To perform the acid pretreatment, 5 ml of 1% sulphuric acid was added to 10 g of grinded powder and heated at 80 °C for 20 min. The acid pretreated solution was then allowed to reach room temperature so that the lignocellulosic mass can be obtained. Then, 100 ml of 4% sodium hydroxide was added in the cool down acid pretreated solution and boiled for 30 min. The solution was then filtered and autoclaved and finally stored in the freeze for future use as stock solution [28].

#### Isolation of bacteria using alkali lignin

The lignin-degrading bacteria were isolated using lignin enriched minimal salt medium (MSM-L) in which previously prepared alkali lignin served as the sole carbon and energy source. To prepare 1% alkali lignin solution, 1 ml of previously prepared stock solution (Alkali lignin) was added with 99 ml of distilled water. MSM-L consisted of 1% alkaline lignin minimum salt medium solution which contained (g/L): K_2_HPO_4_, 4.55; KH_2_PO_4_, 0.53; MgSO_4_, 0.5; and NH_4_NO_3_, 5 [29, 30]. Enriched culture were prepared in a 250-ml Erlenmeyer flask by placing 5 g sample in 95 ml of autoclaved MSM-L, and the culture were incubated at 30 to 45 °C at 120 rpm for 7 days. One milliliters enriched sample was transferred to 99 ml of sterile water. Using 1 ml of the liquid mixture, serial dilution technique was performed from each dilution. About 100 μl of serially diluted 10^−6^ sample were spread on plate containing MSM-L. The plates were incubated at 30 °C for 7 days until colonies developed. The isolated bacteria were plated onto fresh MSM-L agar plates repeatedly to obtain pure cultures [31]. Different sub colony was selected according to morphological characteristic like shape, size, and colour. Morphologically isolated and distinguished bacteria colony from the pour plate of each soil sample was transferred to MSM-L agar medium and incubated at 37 °C for 3 days. This method was repeated until single and pure bacterial culture was obtained [28]. Pure bacterial cultures were designated as Simlipal Ligninolytic Bacteria (SLB), and for the convenience of work, all bacterial isolates were named in abbreviated form as SLB1, SLB2, SLB3 up to the total number of occurrence for all future contexts.

### Screening of ligninolytic bacteria

#### Qualitative assay of ligninolytic activity

The bacterial isolates were screened using methylene blue dye as an indicator. The bacteria possessing ligninolytic enzymes undergo oxidation of the indicator dye. Methylene blue is a phenolic compound and retains the homologous chemical structure like the subunits found in lignin polymer. Ten microliters (6.2 × 10^9^ CFU/ml) of each bacterial broth was inoculated inside a 4-mm pore made on the Luria broth (LB) agar plate containing methylene blue dye. The LB agar medium contains (g/L) NaCl: 1, tryptone: 2, yeast extract: 1, agar: 2, and methylene blue dye: 0.25. Ten microliters distilled water was used as a control in the LB agar plate. The plates with the bacterial inoculums were incubated at 30 °C for 72 h. The agar plates were monitored daily for bacterial growth and decolourization of methylene blue dyes [32]. Occurrence of any bacterial growth on LB agar plate (colony diameter, C) and appearance of hydrolyzing zone (H) around the bacterial growth due to the decolourization of methylene blue dye was monitored daily to determine the ligninolytic activity by measuring its diameter. For the determination of H to C value, the ratio of hydrolyzing zone and colony diameter were measured individually for each bacterial strain.

#### Quantitative assay of ligninolytic activity

Quantitative assay was carried out to quantify the decolourization of lignin broth which indicates the growth of ligninolytic bacteria in the lignin broth. Bacterial isolates that have shown highest ligninolytic activity in the screening using methylene blue indicator were taken in this quantitative assay. All the selected isolates for the first phase of quantitative assay were freshly inoculated in 50 ml of 0.5% lignin broth in a 250-ml flask, and an un-inoculated control was also kept. All the flasks were incubated at 30 °C at 120 rpm for 7 days in a shaker incubator, and absorbance was recorded on daily up to 7 days at a wavelength of 465 nm in UV-Vis spectrophotometer (Systronic-119) [33]. All the experiments have been performed in triplicates. The percentage of decolourization for each isolate was calculated using the Eq. 1:

1 $$\mathrm{Percentage}\ \mathrm{of}\ \mathrm{decolourization}=\frac{\left(\mathrm{A}465\ \mathrm{on}\ 1\mathrm{st}\ \mathrm{day}-\mathrm{A}465\ \mathrm{on}\ 7\mathrm{th}\ \mathrm{day}\right)}{\mathrm{A}465\ \mathrm{of}\ \mathrm{control}\ \mathrm{on}\ 7\mathrm{th}\ \mathrm{day}}\times 100$$

A465 = Absorbance at wavelength 465 nm.

### LiP enzyme assay

The isolates (SLB8, SLB9, and SLB10) that showed significant percentage of decolourization in the quantitative assay were further selected for the lignin peroxidase enzyme assay. LiP activity can be determined on the basis of demethylation of methylene blue dye. Methylene blue serves as an indicator in this enzyme assay, and the enzyme LiP demethylates methylene blue in the presence of H_2_O_2_. The final product is a tri-demethylated methylene blue derivative known as azure C, and the reaction occurs at pH 4. Enzyme activity was determined as per the percent decolourization of the methylene blue dye. About 1 ml of culture broth of each isolates were taken in the Eppendorf tubes, and the culture broths were centrifuged at 4 °C at 7000 rpm in cooling centrifuge. The supernatant from each centrifuge tubes were then collected for conducting the enzyme assay as the lignin peroxidase is an extracellular protein. In 1 ml of 50 mM sodium potassium tartarate (pH -4) buffer, 0.1 ml of 0.1 mM H_2_O_2_ inducer was added. Then to this solution, 32 μM methylene blue and 10 μl of enzyme supernatant were added. In this assay, 10 μl of distilled water was added with the above reaction mixture in place of enzyme supernatant in a separate test tube as control. The final assay solution was incubated for 1 h at room temperature and absorbance was measured in UV-Vis spectrophotometer (Systronic-119) at a wavelength of 650 nm [34]. All the experiments have been done in triplicates. The results were elucidated as the percentage decolourization of methylene blue dye by the enzyme LiP in comparison with the control tube determined using the Eq. 2:

2 $$\frac{\left(\mathrm{A}650\ \mathrm{control}-\mathrm{A}650\ \mathrm{for}\ \mathrm{test}\right)}{\mathrm{A}650\mathrm{for}\ \mathrm{control}}\times 100$$

A650 = Absorbance at wavelength 650 nm.

The most efficient ligninolytic bacterial strain obtained after the LiP enzyme assay would further be taken for all other experimental analyses like growth curve analysis; morphological, biochemical, and molecular identification; and optimization of bacterial LiP activity.

### Growth curve of bacterial isolate

The bacterial population was calibrated after specific time intervals, and the number of viable bacteria is plotted in graph with respect to time, which gives a logarithmic growth curve. Overnight grown culture of 2 ml (10^4^ cell/ml according to the dilution factor) of the ligninolytic bacterial isolate was inoculated in a 200 ml of sterilized lignin broth at 37 °C for 42 h with continuous shaking. To obtain the growth curve in terms of viable cell counts, culture suspension was taken at a time intervals of 2 h followed by serial dilution and plating of aliquots to determine the CFU/ml.

### Identification of ligninolytic bacterium

#### Morphological characterization

Morphological characterization includes the study about the morphology (size, shape, and texture) of the bacteria. To study different morphological features, Gram’s stain of the ligninolytic bacterium was performed following the protocol as mentioned by Coico [35]. The bacterium was examined with a phase contrast microscope (×100 objectives) after Gram’s staining.

#### Biochemical characterization of ligninolytic bacterium

The ligninolytic bacterium was further considered to perform different biochemical tests as listed in the Bergey’s manual of Systematic Bacteriology for its identification purpose [36].

#### Molecular identification of ligninolytic bacterium

Molecular identification of ligninolytic bacterial strain was carried out by 16S rRNA gene sequencing. DNA of the bacterium was extracted, and the 16S rRNA gene was amplified based on the standard methods using the universal primers [37]. Purification of the amplified 16S rRNA gene was performed and sent to Applied Bioscience Eurofins, Bangalore, for its sequencing.

##### Preparation of phylogenetic tree

To prepare the phylogeny tree of the bacterium, the construction of the contig 16S rRNA gene is required. Contig sequence of 16S rRNA gene of the ligninolytic bacterium was constructed with the help of CAP3 webserver [38]. In this purpose, the forward and reverse sequence information of that bacterium retrieved from 16S rRNA gene sequencing were used. Other closely related homologues sequences of 16S rRNA genes with the contig sequence of the ligninolytic bacterium were retrieved from the nucleotide BLAST search [39]. The contig sequences along with all the retrieved sequences from BLAST search was compared both by the pairwise and multiple sequence alignment in ClustalW tool. Neighbour-joining (NJ) method was applied to prepare the phylogenetic tree for the ligninolytic bacterium in MEGA 7.0.26 (Molecular Evolutionary Genetics Analysis) software [40] using the output alignment file obtained from ClustalW. Percentage of homogeneity between the associated taxa containing the bacteria clustered together was determined by bootstrap test [41]. Maximum composite likelihood (MCL) method was used to calculate the evolutionary distances in phylogenetic tree [42].

### Assessment of carbon, nitrogen, and metal sources in regulating lignin peroxidase activity

Selection of preferable carbon (glucose, fructose, starch, and cellulose) and nitrogen (tryptone, urea, peptone, ammonium nitrate, sodium nitrate, and yeast extract) sources that promote lignin peroxidase activity in the ligninolytic bacterium was done by culturing the bacterium separately in medium supplemented with different carbon and nitrogen sources at a concentration of 0.4% (w/v). Different sources of metal ions (KCl, MgSO_4_, MnSO_4_, ZnSO_4_, CuSO_4_, FeSO_4_, NaCl, CaCl_2_, CoCl_2_) were also individually applied to the bacterial growth medium at a concentration of 0.1% (w/v) to analyze their impact in enzyme activity [43, 44]. All the experiments in the above mentioned chemical optimization study have been performed in triplicates.

### Assessment of carbon, nitrogen, and metal sources in dye decolourization by lignin peroxidase

Dye decolourizing ability by the LiP enzyme from the ligninolytic bacterium was performed by the quantitative assay as mentioned previously. In this purpose, the basal medium used for culturing the ligninolytic bacterium was individually supplemented by respective carbon, nitrogen, and metal ion sources as mentioned above to measure the enzyme activity.

### Optimization of lignin peroxidase enzyme activity

#### Experiment design and statistical optimization of lignin peroxidase activity using response surface methodology (RSM)

To study the combinatorial impact of the variables viz. incubation time, pH, substrate concentration, and temperature on the activity of lignin peroxidase (LiP) enzyme, the Box-Behnken design was employed. Table 1 represents the range and three specific levels of the above mentioned variables that were optimized for maximizing the LiP activity. The low and high levels of each independent variable were marked as − 1 and + 1, respectively, whereas the center level was coded as 0 [45]. The Box-Behnken design is preferable for the construction of both the quadratic response surfaces and a second-degree polynomial model. This second-degree polynomial model is applied to optimize a process in case of a small number of experimental runs [46]. The design of experimental models was performed using the Design-Expert (Version 12, Stat-Ease Inc., Minitab Inc., USA) statistical software on the basis of a second-degree polynomial equation which resulted 27 experimental runs. The second-degree polynomial is demonstrated as Eq. 3:

**Table 1:** Actual levels for the four variables Box-Behnken design

Independent variables	Symbols	Coded and actual levels		
		− 1(low)	0	+ 1(high)
Incubation time (h)	A	12	42	72
pH	B	5.5	8.0	10.5
Substrate conc (mM)	C	0.030	0.115	0.200
Temperature (°C)	D	30	37.5	45

3 $$\boldsymbol{Y}={\boldsymbol{b}}_{\mathbf{0}}+{\boldsymbol{b}}_{\mathbf{1}}\boldsymbol{A}+{\boldsymbol{b}}_{\mathbf{2}}\boldsymbol{B}+{\boldsymbol{b}}_{\mathbf{3}}\boldsymbol{C}+{\boldsymbol{b}}_{\mathbf{4}}\boldsymbol{D}+{\boldsymbol{b}}_{\mathbf{12}}\boldsymbol{A}\boldsymbol{B}+{\boldsymbol{b}}_{\mathbf{13}}\boldsymbol{A}\boldsymbol{C}+{\boldsymbol{b}}_{\mathbf{23}}\boldsymbol{B}\boldsymbol{C}+{\boldsymbol{b}}_{\mathbf{24}}\boldsymbol{B}\boldsymbol{D}+{\boldsymbol{b}}_{\mathbf{11}}{\boldsymbol{A}}^{\mathbf{2}}+{\boldsymbol{b}}_{\mathbf{22}}{\boldsymbol{B}}^{\mathbf{2}}+{\boldsymbol{b}}_{\mathbf{33}}{\boldsymbol{C}}^{\mathbf{2}}+{\boldsymbol{b}}_{\mathbf{44}}{\boldsymbol{D}}^{\mathbf{2}}$$

In the above equation, *Y* represents the estimated response which actually depicts the lignin peroxidase (LiP) activity. In the present study, four factors *viz*, incubation time (*A*) [12, 42, and 72 (h)]; pH (*B*) [5.5, 8.0, and 10.5]; substrate concentration (*C*) [0.030, 0.200, and 0.115 (mM)], and temperature (*D*) [30, 37.5, and 45 (°C)] were considered in the Box-Behnken design for measuring the LiP enzyme activity from the ligninolytic bacterium. In the second-degree polynomial equation mentioned above, *b*_0_ indicates the inter shape or offset term; *b*_1_, *b*_2_, *b*_3_, and *b*_4_ are the terms responsible for linear affect; and *b*_11_, *b*_22_, *b*_33_, and *b*_44_ are interaction terms during the calculation of LiP activity. According to above mentioned equation, the RSM data were further subjected to regression analysis of variance to fit the model. Fit statistics of the response model for LiP activity of the ligninolytic bacterium was calculated. The adequacy of the resulting model was verified by model analysis and *R*^2^ analysis. *F* value was determined to check the statistical significance of the entire calculated model at 5% level of significance [47]. For the determination of the optimum condition for the highest lignin peroxidase activity, the fitted equation was presented as 3D surface plots, which is also constructed by the Design-Expert software.

#### Culture of bacterium as per the experiment designed

On the basis of experimental conditions designed in RSM, bacterial culture was made in a 250-ml Erlenmeyer flask containing culture broth MSM-L medium. The medium was composed of (g/L) K_2_HPO_4_, 4.55; KH_2_PO_4_, 0.53; MgSO_4_, 7H_2_O, 0.5; and NH_4_NO_3_, 5 supplemented with 1% alkali lignin. The pH of the culture broth was set to 3 different variables (5.5, 8.0, and 10.5). All the flasks filled with MSM-L culture broth were autoclaved followed by the addition of different concentrations (g/L) of H_2_O_2_ (0.030, 0.115, and 0.200) as a substrate to measure LiP activity. Each flask was then inoculated with 0.5% (31 × 10^11^ CFU/ml) of standard inoculum (v/v) of the ligninolytic bacterial strain. The inoculated flasks were then placed on a rotary shaker incubator with a shaking speed of 120 rpm to incubate the bacterial culture at specific temperature (30 °C, 37.5 °C, and 45 °C) and for specific time (12, 42, and 72 h) as mentioned in the experimental design in RSM.

#### Quantitative estimation of LiP activity

After the completion of incubation as mentioned above, incubated bacterial culture broth with a volume of 1 ml from each flask was drawn in a separate Eppendorf tube for a specific experimental condition. All the Eppendorf tubes with 1 ml of bacterial culture incubated under specific experimental condition were immediately transferred and kept inside an ice box to avoid the denaturation of enzyme. In the present study, LiP activity was calibrated in triplicate for each experimental condition. All Eppendorf tubes filled with 1 ml of bacterial culture broth were centrifuged at 7000 rpm for 10 min at 4 °C. After the completion of centrifugation, the supernatants from each Eppendorf tube were collected to conduct the LiP enzyme assay for the estimation of LiP activity [48]. Triplicate samples were used during the LiP activity estimation for each experimental condition as mentioned in the RSM. The LiP activity calculated from each experimental condition was then taken for the preparation of 3D surface plots and carrying out of the regression analysis and analysis of variance (ANOVA).

## Results

### Isolation of ligninolytic bacteria

**Fig. 1:**
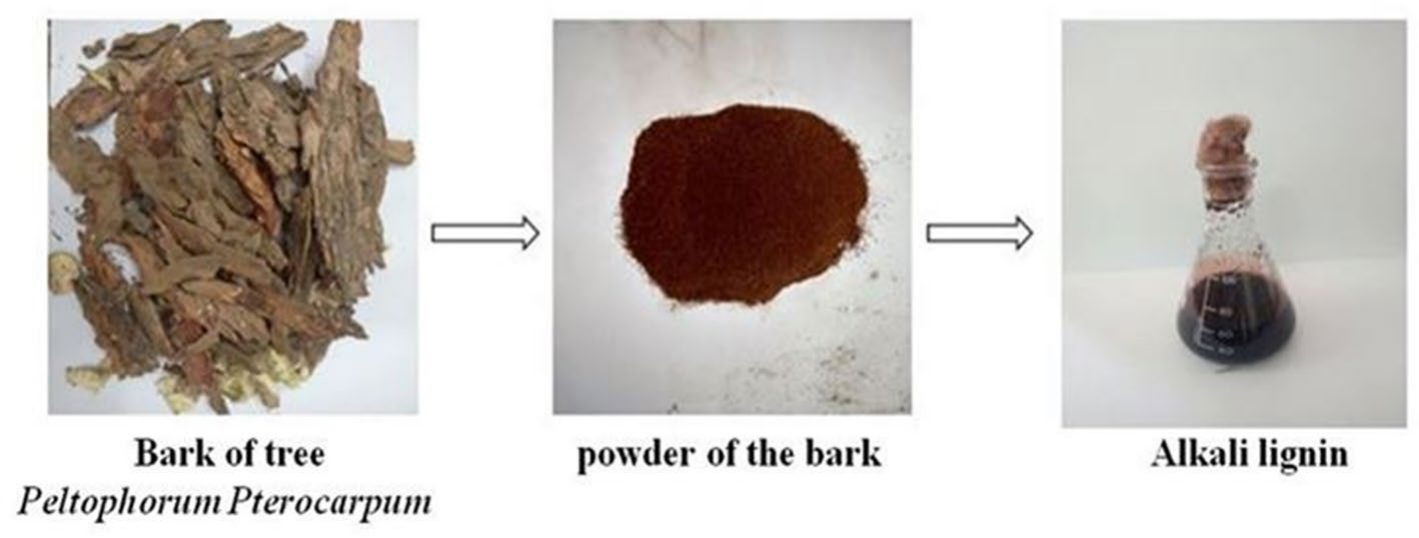
Preparation of alkaline lignin

In this study, a dark brown coloured homogeneous solution of alkali lignin was prepared (Fig. 1). Enriched minimum salt medium containing the freshly prepared alkali lignin (MSM-L) was used for the growth of ligninolytic bacterial colonies in the pour plates. After incubating the plate at 37 °C for 72 h, different bacterial colonies were obtained using morphological characteristics like size, shape, margin, and colour. A total of 16 ligninolytic bacterial colonies were isolated using pour plate method after serial dilution, from the rhizospheric soil samples collected from SBR and were named as SLB1 to SLB16.

### Screening of ligninolytic bacteria

**Fig. 2:**
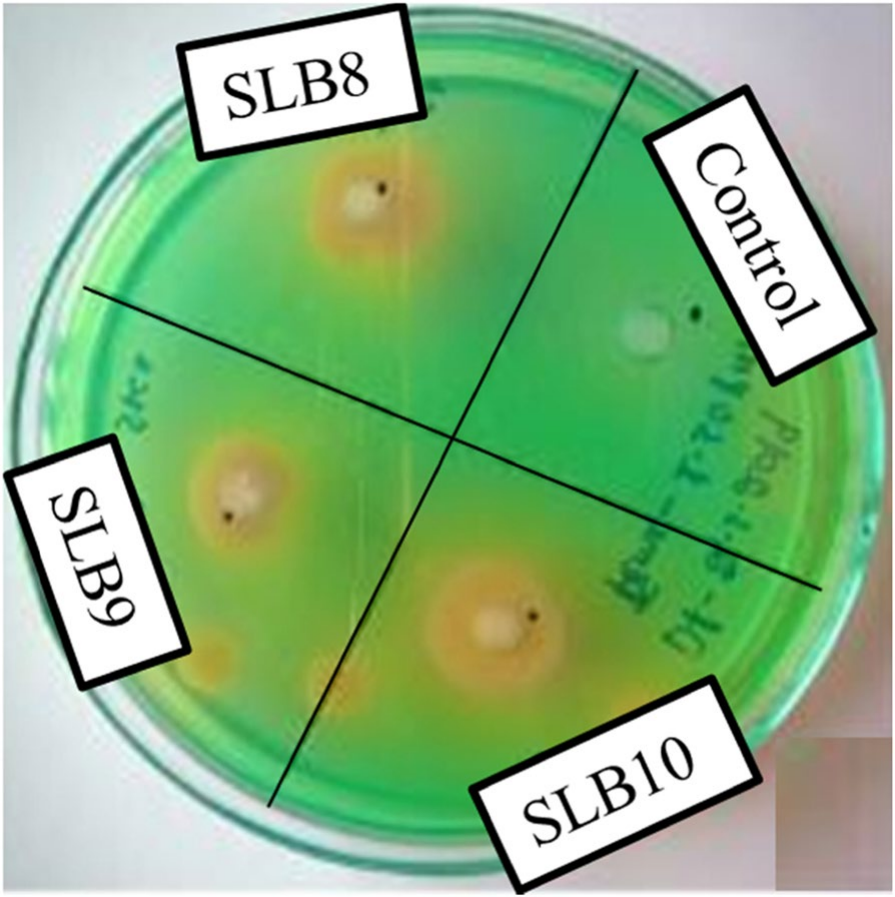
Screening of ligninolytic bacterial isolates on LB agar medium containing methylene blue dye showing zone of decolourization for the bacterial isolates SLB8, SLB9, and SLB10

All the 16 bacterial isolates were primarily screened for ligninolytic activity using methylene blue dye as an indicator. The bacteria possessing ligninolytic enzymes are able to oxidize the indicator dye methylene blue and decolourize the dye (Fig. 2). Among the 16 ligninolytic bacterial strains isolated from the soil samples, 9 isolates showed ligninolytic activity which was measured as per the H to C value (H—hydrolyzing zone, C—colony diameter). Comparative H to C values showed the maximum decolourization zone diameter of the bacterial strain SLB10 with a value of 1.38 followed by SLB9, SLB8, and SLB1 with H to C values of 1.36, 1.33, and 1.21, respectively. The range of the H to C values recorded for the ligninolytic bacteria was between 1.05 and 1.38.

#### Quantitative assay for ligninolytic activity

From the primary screening, three bacterial strains (SLB8, SLB9, and SLB10) which showed the most H to C ratio among all were selected for the quantitative assay for ligninolytic activity. Absorbance values of the lignin containing culture broth inoculated with those ligninolytic bacteria were measured at a daily interval for 7 days after the inoculation. Among all these 3 isolates, SLB8 showed the highest absorbance of 0.781 at day 1 which gradually decreases to 0.649 at day 7. SLB9 showed comparatively less absorbance value of 0.752 at day 1 which decline to 0.609 at day 7. Whereas, strain SLB10 presented the minimum absorbance of 0.745 at day 1 among all these 3 isolates which decreased to the lowest absorbance value of 0.571 at day 7. In reference with the absorbance value (0.851) of the control (un-inoculated lignin broth), the percentage of decolourization was measured for each isolates. The result exhibited that SLB10 has the highest lignin decolourization efficiency with a value of 33.02% compared to the other two isolates which has a decolourization percentage of 15.51% and 16.80% for SLB8 and SLB9, respectively.

### Enzyme assay for lignin peroxidase

**Fig. 3:**
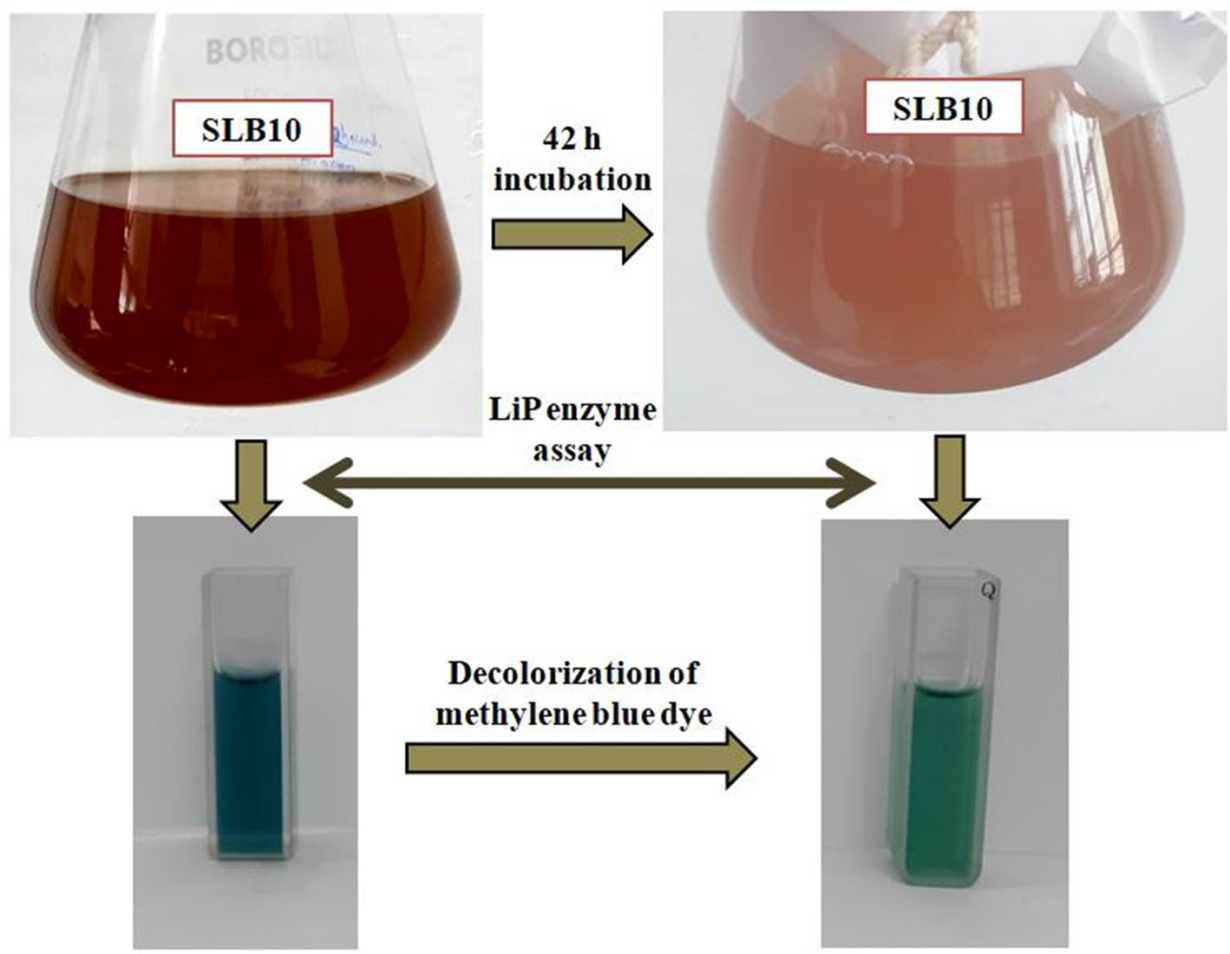
Growth of the bacterium SLB10 in the culture flask containing MSM-L medium and the cuvettes showing the pattern of dye decolonization of methylene blue during LiP enzyme assay

The enzyme lignin peroxidase demethylates methylene blue in the presence of H_2_O_2_. According to the result obtained from the primary screening, 3 bacterial isolates *viz.* SLB8, SLB9, and SLB10 were selected for the lignin peroxidase enzyme assay. From this enzyme assay and in the aspect of % decolourization of the dye methylene blue, the isolate SLB10 showed the highest activity with a value of 31.711 U/mg (Fig. 3). The other isolates, SLB8 and SLB9, showed comparatively lower lignin peroxidase activity with the values of 13.590 U/mg and 16.609 U/mg, respectively.

### Growth curve analysis for SLB10 isolate

**Fig. 4:**
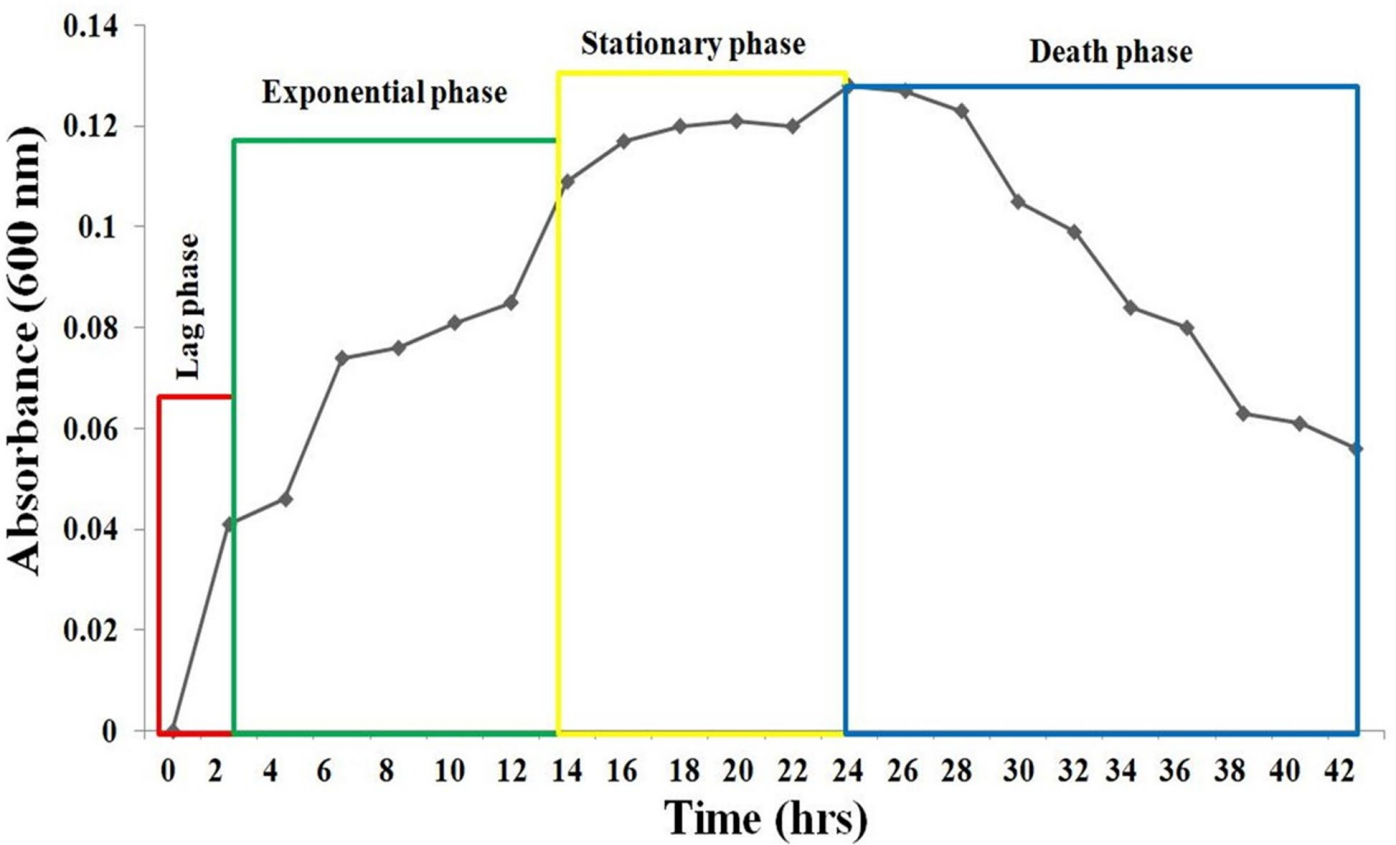
Growth curve of the bacterial strain SLB10

The changes in the number of bacterial population over time for the isolate SLB10 is obtained from a logarithmic growth curve (Fig. 4). It can be observed from the logarithmic growth curve that the lag phase of the bacterium lasts still 4 h after the incubation. At the end of the lag phase, the log_10_ CFU/ml value of culture medium was recorded as 6.8389. The exponential phase of the bacterium was continued after the 4 h and lasted for just before 16 h of the incubation with a change of log_10_ CFU/ml value from 6.8389 to 7.2443. The stationary phase of the bacterium was obtained between the 16 and 24 h of the incubation and the log_10_ CFU/ml value ranges from 7.2443 to 7.2833. The death phase shown to be started after 24 h of incubation, and at the end of 42 h of incubation, the log_10_ CFU/ml value declined near 6.9243.

### Morphological and biochemical characterization of SLB10 isolate

In morphological characterization, texture of the bacterial colony, shape of the bacterial cell, and the pattern of gram staining of the bacterium were identified. The colony of SLB10 bacterium appeared to be in a form of elongated hairy with characteristic swirls. The bacterium was shown to have a rod-shaped structure and gram-positive characteristics when observed under microscope followed by staining procedure. Different biochemical tests were also conducted to obtain more information regarding the identification of the ligninolytic strain SLB10. The bacterium SLB10 exhibited a positive response in urease test as the colour of the medium changes to pinkish red after the bacterial inoculation. In both the oxidase and catalase tests, the SLB10 bacterium showed negative reaction as the colour of the broth remain unchanged after a few seconds and there is no formation of gas with white bubble as in case of oxidase and catalase test respectively. In methyl red test, the bacterium SLB10 gave a positive indication by transforming the colour of the growth medium in red. The strain SLB10 exhibited a positive result in Voges-Proskauer test as the broth developed a crimson yellow colour. In carbohydrate metabolism test, a positive indication has been observed for the bacterium SLB10 as the colour of the growth medium changed to yellow with the formation of gas. In case of both the citrate utilization test and triple sugar iron test, the bacterium SLB10 presented negative response as there is change of bacterial agar slant colour into blue and red in case of citrate utilization test and triple sugar iron test, respectively. As per the results obtained from all the morphological and different biochemical tests and references from Bergey’s manual of Systematic Bacteriology, the ligninolytic bacterium SLB10 was primarily identified as a member belonging from the genus *Bacillus*.

### Molecular identification of SLB10 isolate

Result obtained from the analysis of 16S rRNA gene sequencing has shown that the ligninolytic bacterium SLB10 has forward and reverse partial sequences with a length of 946 and 755 bp, respectively.

#### Analysis of phylogenetic tree for SLB10 isolate

**Fig. 5:**
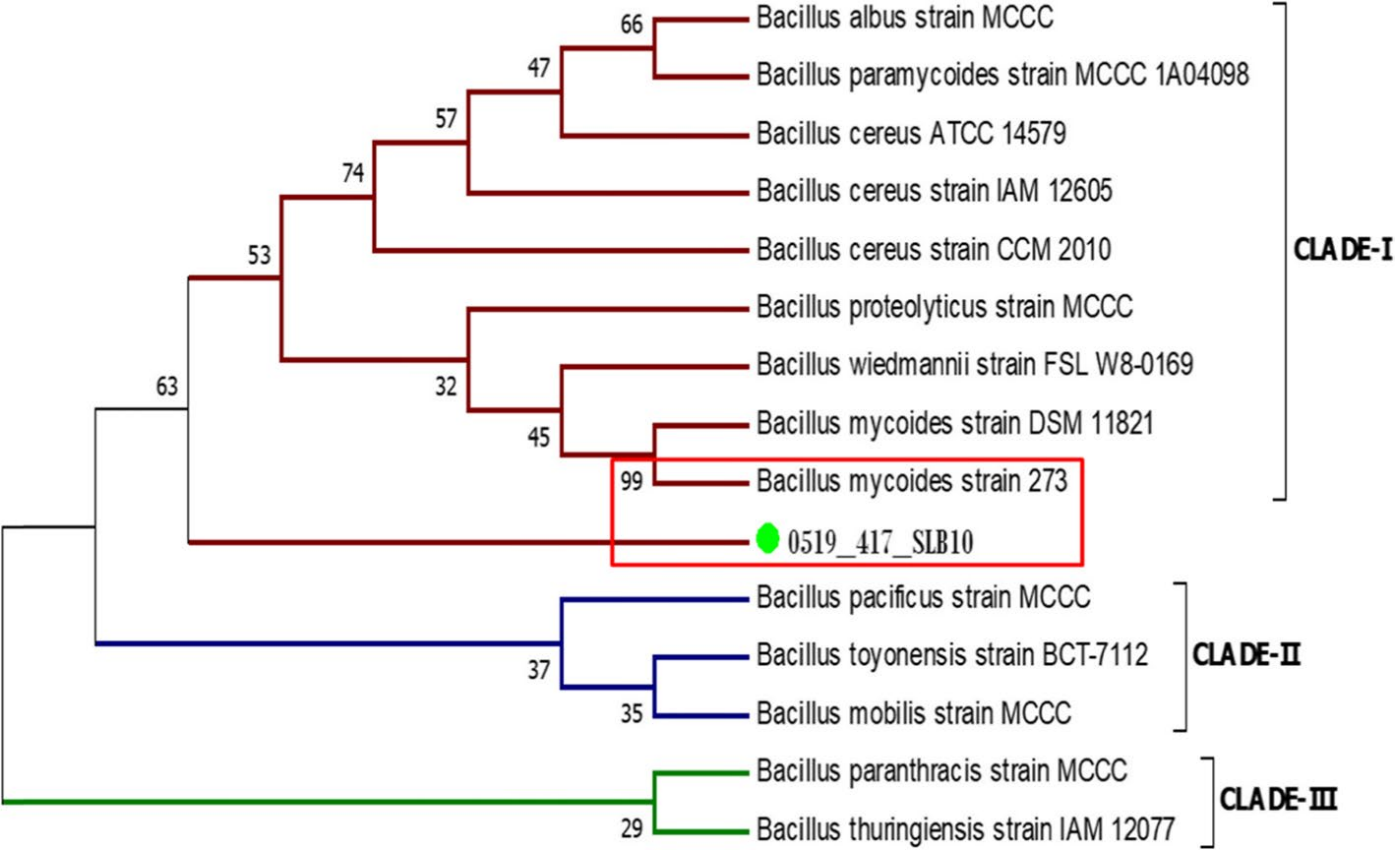
Phylogenetic tree of the ligninolytic strain SLB10 based on 16S rRNA sequence

Neighbour-Joining method was carried out to prepare the phylogenetic tree with the help of 16S rRNA sequence information of SLB10 for the species level identification of the bacterium. According to the result of phylogenetic analysis, it has been found that the 16S rRNA gene sequence alignment of isolate SLB10 is intimately linked with *Bacillus mycoides* strain 273 with a percentage of homogeneity value of 99 which is the highest among all compared (Fig. 5). From the phylogeny tree, it can be also observed that due to the lowest evolutionary distance, the tested ligninolytic bacterium SLB10 and the closed homologues *Bacillus mycoides* strain 273 are clustered together under the CLADE-I. Therefore, based on the analysis from 16S rRNA gene sequencing and phylogeny tree, the SLB10 strain was recognized as *Bacillus mycoides*.

### Optimization of lignin peroxidase enzyme activity

For the optimum bacterial growth and its enzyme function, various inorganic nutrients such as carbon, nitrogen, and metal ion sources are necessary. Therefore, experiments have been carried out to determine the impact of carbon, nitrogen, and metal ions on the lignin peroxidase activity of the bacterium *Bacillus mycoides*. Effect of these carbon, nitrogen, and metal ion sources on the ability of dye decolourization mediated by lignin peroxidase (LiP) of *B. mycoides* has also been determined. In addition to maximize the LiP activity of *B. mycoides*, optimization of multiple parameters including pH, temperature, substrate concentration, and incubation time during the bacterial growth was performed on the basis of experiments designed by response surface methodology (RSM).

#### Effect of carbon sources on LiP activity and dye-decolourization

**Fig. 6:**
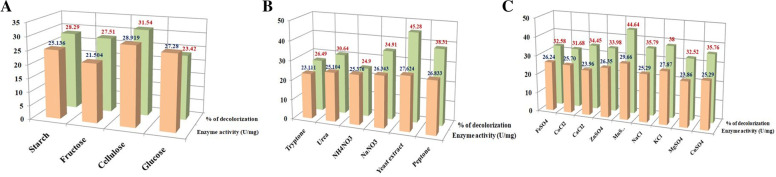
Effect of different carbon sources (**a**), nitrogen sources (**b**), and metal ion sources (**c**) on LiP activity and dye decolourization by the bacterium *Bacillus mycoides*

**Fig. 7:**
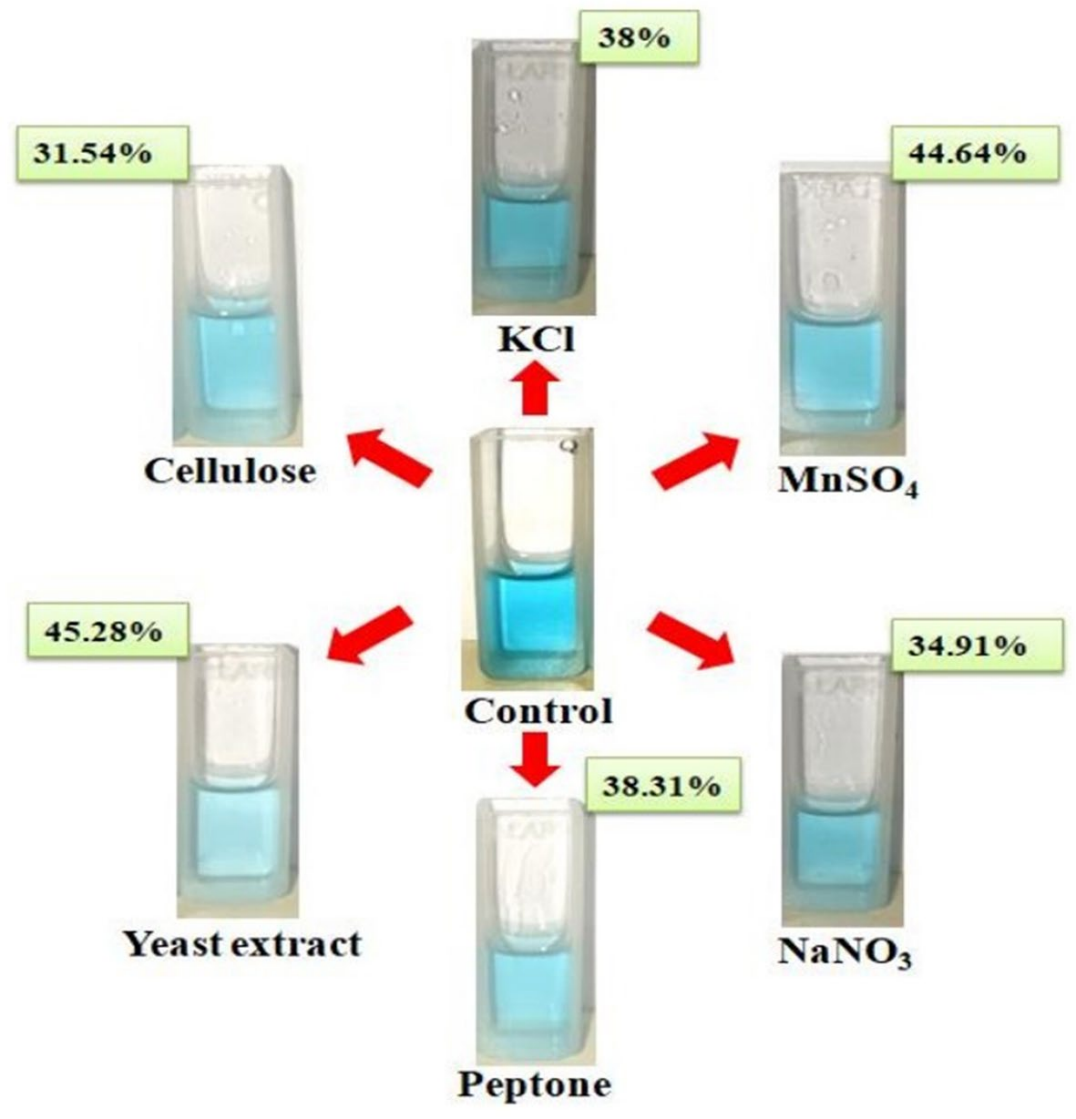
Dye-decolourization pattern for the enzyme LiP from *Bacillus mycoides* cultured in different enriched media supplemented with cellulose, KCl, MnSO_4_, peptone, NaNO_3_, and yeast extract. The percentage values indicate degree of dye decolourization in respective enriched media supplemented with specific carbon, nitrone, and meal ion sources

The effect of various carbon sources on LiP activity of *B. mycoides* was carried out in the same basal medium and culture conditions. Analysis the result from this experiment depicted that highest LiP activity of 28.919 U/mg has been recorded in the presence of cellulose in basal medium in comparison with the presence of other three carbon sources *viz.* starch (25.136 U/mg), fructose (21.504 U/mg), and glucose (27.280 U/mg) (Fig. 6). This result implied that cellulose might have some inducing effect in promoting lignin peroxidase activity. In terms of percentage of dye decolourization for different carbon sources, it has been found that highest decolourization of 31.54% was recorded in case of cellulose supplemented medium for *B. mycoides* (Figs. 6 and 7). Followed by cellulose other three carbon sources *viz.* starch, fructose, and glucose supplemented bacterial growth medium showed dye-decolourization percentage of 28.29, 27.51, and 23.42%, respectively (Fig. 6).

#### Effect of nitrogen sources on LiP activity and dye decolourization

The function of the enzyme is also greatly influenced by one of the key causes, which is a separate source of nitrogen. In this study, maximum LiP activity has been found in case of yeast extract supplemented basal medium with a value of 27.624 U/mg followed by other two nitrogen sources, peptone (26.833 U/mg) and sodium nitrate (26.343 U/mg), respectively. Comparatively, reduced enzymatic activity has been recorded for the nitrogen sources *viz.* tryptone (23.111 U/mg), urea (25.104 U/mg), and NH_4_NO_3_ (25.376 U/mg) when these were presence in the basal medium for bacterial culture (Fig. 6). Likewise, highest LiP activity in yeast extract supplemented bacterial growth medium a maximum dye-decolourization ability of 45.28% has been recorded. Peptone supplemented bacterial growth medium showed the second highest dye-decolourizing efficacy with a percentage of 38.31 (Figs. 6 and 7). Other four nitrogen sources supplemented medium showed dye-decolourization efficiency ranges between 24.9 and 34.91% (Fig. 6).

#### Effect of metal ion sources on LiP activity and dye decolourization

Different metal ion sources have shown major effect in the variation of LiP activity. From the analysis of present study, it has been implied that the presence of MnSO_4_ and KCl in the basal medium of growth for *B. mycoides* resulted in comparatively higher LiP activity with the values of 29.659 U/mg and 27.872 U/mg, respectively. Other tested metal ion sources has more or less similar impact on LiP activity which has been reflected by the range of enzyme activity value from 23.862 U/mg to 26.236 U/mg (Fig. 6). Presence of the metal ion source MnSO_4_ in bacterial growth medium showed the highest dye-decolourizing percentage of 44.64 compared to all other tested metal ion sources (Figs. 6 and 7).

#### Optimization of growth factors using RSM

RSM was applied to detect the response of LiP activity for the ligninolytic bacterium *Bacillus mycoides* to various growth factors as mentioned above using Box-Behnken design. The full experimental plan as per the designed experiment condition along with the response values are given in the Table 2.

**Table 2:** Optimization of fermentation parameters using response surface methodology with Box-Behnken design

Conditions	pH	Temperature (°C)	Incubation time (h)	H_2_O_2_ conc. (mM)	LiP activity experimental (U/mg)	LiP activity predicted (U/mg)
1	5.5	45.0	42	0.115	28.241	27.563
2	8.0	37.5	72	0.200	19.649	19.656
3	8.0	45.0	42	0.200	37.155	36.369
4	5.5	30.0	42	0.115	7.183	6.369
5	8.0	37.5	72	0.030	16.631	15.369
6	10.5	37.5	42	0.030	27.710	26.970
7	8.0	30.0	42	0.200	9.400	9.633
8	10.5	37.5	72	0.115	27.710	25.636
9	8.0	30.0	72	0.115	13.596	13.756
10	5.5	37.5	12	0.115	21.175	22.365
11	10.5	37.5	12	0.115	34.189	40.220
12	5.5	37.5	42	0.200	33.263	33.696
13	10.5	45.0	42	0.115	30.240	30.256
14	8.0	45.0	72	0.115	20.345	21.365
15	5.5	37.5	42	0.030	39.315	39.698
16	8.0	37.5	12	0.200	45.368	45.365
17	10.5	30.0	42	0.115	55.947	54.639
18	10.5	37.5	42	0.200	37.807	36.546
19	8.0	30.0	42	0.030	33.263	33.658
20	8.0	37.5	42	0.115	27.714	26.970
21	8.0	45.0	42	0.030	13.135	13.750
22	8.0	37.5	42	0.115	25.483	26.369
23	5.5	37.5	72	0.115	25.702	25.639
24	8.0	30.0	12	0.115	31.206	30.569
25	8.0	45.0	12	0.115	16.630	16.840
26	8.0	37.5	12	0.030	37.155	36.548
27	8.0	37.5	42	0.115	24.789	25.658

##### Regression analysis

The second-order polynomial equation (Eq. 4) obtained from multiple regression analysis shown to explain LiP activity of the ligninolytic bacterial strain *Bacillus mycoides* looks like as follows:

4 $$\boldsymbol{LiP}\ \boldsymbol{activity}\left(\boldsymbol{Y}\right)=+\mathbf{27.96}-\mathbf{5.03}\ast \boldsymbol{A}+\mathbf{6.01}\ast \boldsymbol{B}+\mathbf{1.23}\ast \boldsymbol{C}-\mathbf{1.30}\ast \boldsymbol{D}-\mathbf{3.56}\ast \boldsymbol{A}\boldsymbol{B}-\mathbf{1.06}\ast \boldsymbol{A}\boldsymbol{C}+\mathbf{4.83}\ast \boldsymbol{A}\boldsymbol{D}+\mathbf{3.68}\ast \boldsymbol{B}\boldsymbol{C}-\mathbf{15.69}\ast \boldsymbol{B}\boldsymbol{D}+\mathbf{11.27}\ast \boldsymbol{C}\boldsymbol{D}+\mathbf{2.50}\ast {\boldsymbol{A}}^{\mathbf{2}}+\mathbf{31.50}\ast {\boldsymbol{B}}^{\mathbf{2}}+\mathbf{9.62}\ast {\boldsymbol{C}}^{\mathbf{2}}-\mathbf{1.56}\ast {\boldsymbol{D}}^{\mathbf{2}}$$

In the above mentioned equation, *Y* indicates the response value of LiP activity of the bacterium *Bacillus mycoides*. *A*, *B*, *C*, and *D* represented the incubation time, pH, substrate (H_2_O_2_) conc., and temperature, respectively. The polynomial equation implied that the terms *A*, *B*, *C*, *D*,* AB*, *AC*, *AD*, *BC*, *BD*, *CD*, *A*^2^, *B*^2^, *C*^2^, and *D*^2^ are significant in the designed experimental model for the expression of LiP activity as the calculated *p* value for those terms are < 0.0001 (Table 3). This result also represents the major impact of these parameters towards the highest coefficient value for LiP activity. The coefficient of variation (CV) or the C.V. % indicates the degree of precision with which the experimental conditions has been compared. A low CV value of 2.52% was calculated from the fit statistics in the response model of enzyme activity in RSM (Table 3).

**Table 3:** Analysis of variance (ANOVA), Fit statistic of response model, and coefficient table for LiP enzyme activity of *Bacillus mycoides*

Analysis of variance (ANOVA)						
Source	Sum of squares	Degree of freedom	Mean square	*F* value	*p* value	
Model	2417.07	10	247.11	55.92	<0.0001	
*A*—Incubation time	303.55	1	303.55	68.69	<0.0001	
*B*—pH	433.32	1	433.32	98.05	<0.0001	
*C*—Substrate conc.	18.12	1	18.12	4.10	0.0599	
*D*—Temperature	20.40	1	20.40	4.62	0.0473	
*AB*	50.77	1	50.77	11.49	0.0037	
*AC*	4.48	1	4.48	1.01	0.3288	
*AD*	93.36	1	93.36	21.13	0.0003	
*BC*	54.06	1	54.06	12.23	0.0030	
*BD*	984.86	1	984.86	222.86	<0.0001	
*CD*	508.14	1	508.14	114.98	<0.0001	
*A*^2^	698.77	1	698.77	87.20	<0.0001	
*B*^2^	448.76	1	448.76	682.5	<0.0001	
*C*^2^	831.56	1	831.56	144.55	<0.0001	
*D*^2^	1.99	1	1.99	0.4321	<0.0001	
Residual	95.23	16	8.08			
Lack of fit	70.37	14	5.03	0.4522	0.9513	
Pure error	0.3398	2	0.1699			
Cor total	2541.78	26				
Fit statistics of the response model						
*R*^2^	Adjusted *R*^2^	Predicted *R*^2^	Adeq precision	C.V. %	Standard deviation	
0.9722	0.9548	0.9073	32.3452	2.52	0.6194	
Coefficient table for lignin peroxidase activity						
Interacting terms	*AB*	*AC*	*AD*	*BC*	*BD*	*CD*
*p* value	0.0037	0.3288	0.0030	0.0030	<0.0001	<0.0001

##### Statistical analysis

It is evident from the obtained result of statistical analysis that the quadratic model calculated by RSM was highly significant, as suggested by the model *F* value (55.92) and low probability value (< 0.0001). The lack of fit *F* value of 0.4522 calculated in ANOVA indicates that this value has no major impact towards the pure error which highlight that the designed experiment is mostly valid. According to the *p* value of ANOVA table, there is a 95.13% chance that a lack of fit *F* value becomes larger because of noise (Table 3). The coefficient of determination (*R*^2^) was measured as 0.9722 in the fit statistics of the response model which indicates an enhanced LiP activity. This value represents that the statistical model in this study can elucidate 97.22% of variability in the response. In accordance with this reported statement, the *R*^2^ value as calculated in this study suggests that the designed experiment appropriately predict the enzyme activity. The predicted *R*^2^ value of 0.9073 has shown to be almost close with the adjusted *R*^2^ value of 0.9548 in the fit statistics of response model (Table 3). This indicates a good agreement between the experimental and predicted values for LiP activity (Table 2). It has also been found that the adjusted *R*^2^ (0.9548) is less than the *R*^2^ value (0.9722) as calculated in the present study (Table 3). Estimated *p* value as derived from the coefficient table was reported as < 0.0001 (Table 3) for the three experimental factors, substrate conc. (*C*), pH (*B*), and temperature (*D*) among all interacting terms which implies the major impact of these three factors towards the highest LiP activity.

##### Interplay among variables

**Fig. 8:**
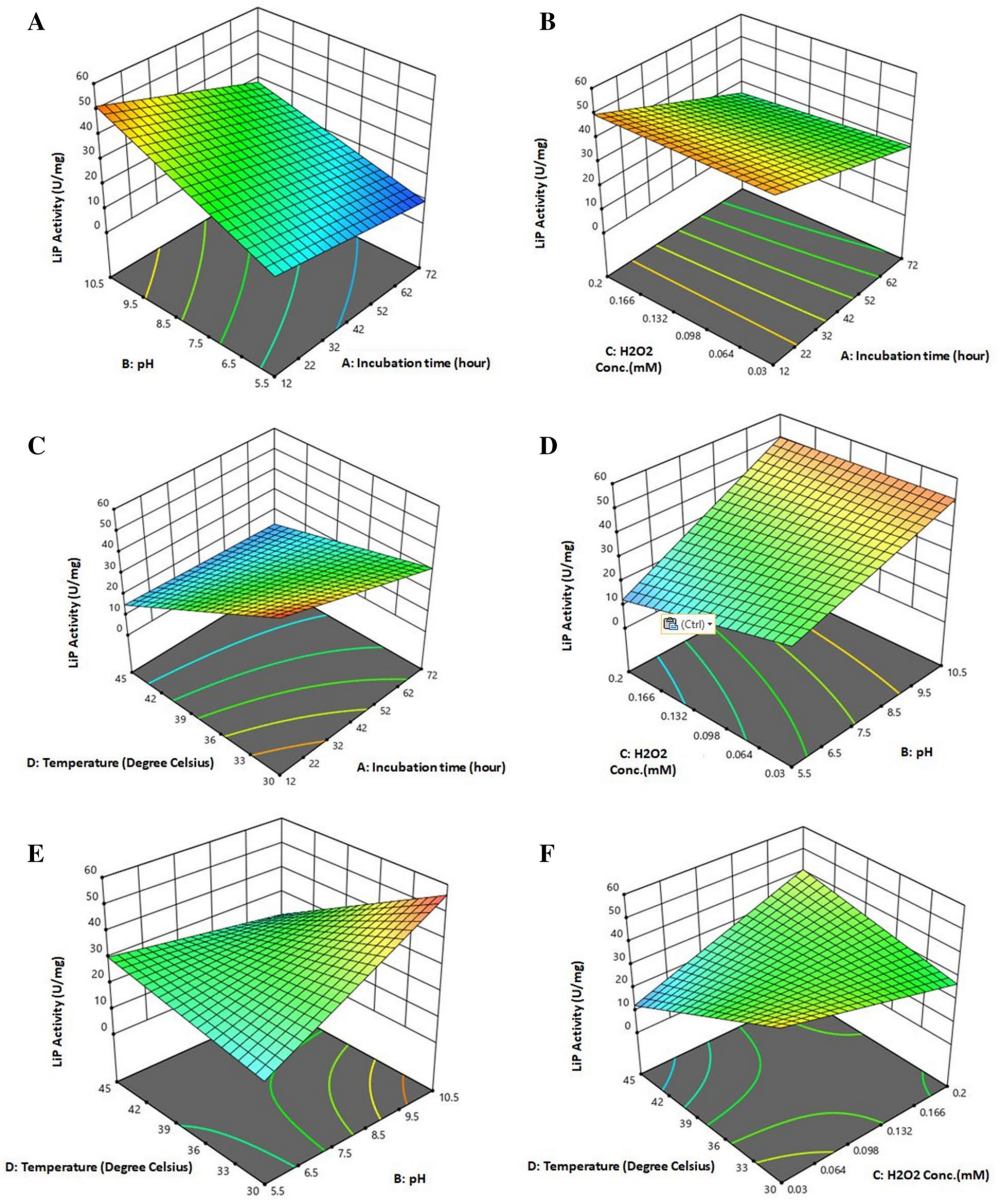
Response surface graph of LiP activity of the ligninolytic bacterial strain *Bacillus mycoides* with respect to different experimental factor. **a** vs. pH, incubation time; **b** vs. substrate concentration, incubation time; **c** vs. temperature, Incubation time; **d** vs. substrate conc., pH; **e** vs. temperature, incubation time; **f** vs. temperature, substrate conc

**Fig. 9:**
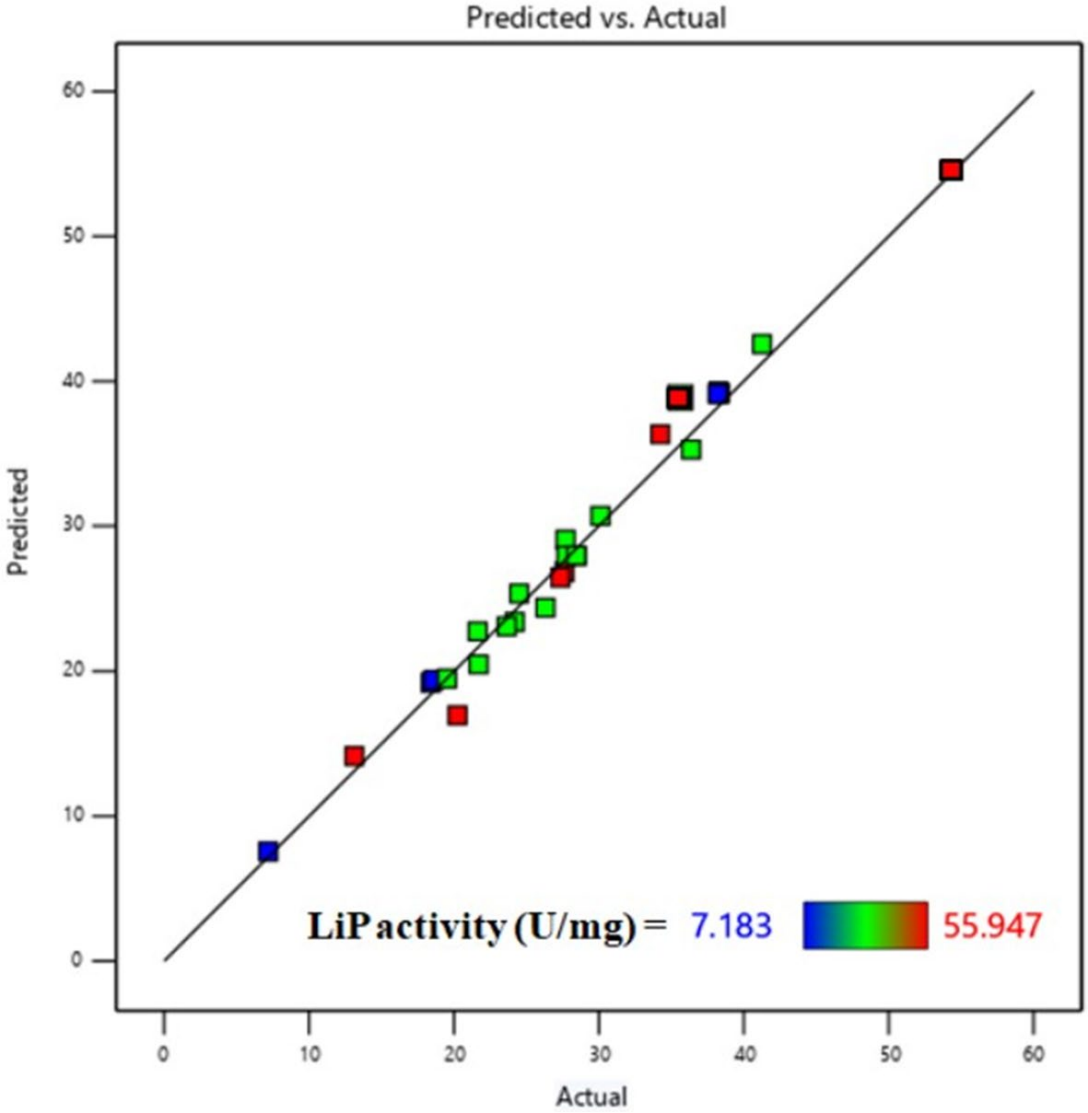
Residual plots for the LiP activity of the ligninolytic bacterial strain *Bacillus mycoides* calculated from RSM

Comparative effects of any two variables on LiP activity of *Bacillus mycoides* were explained by surface plots while holding the other values constant at the point values of different levels. From 3D response surface plots, the interactive effects of experimental factors on LiP enzymes activity were determined. The response surface curves Fig. 8a showed how LiP activity varied with the changes in pH and incubation time while maintaining the levels of temperature and substrate concentration fixed at 30 °C and 0.115 mM, respectively. In this condition, it has been shown that increase in pH resulted in elevation of LiP activity from 7.183 to 55.947 U/mg (Table 2). Figure 8b represented the response surface curves which depicted that there was high LiP activity of 45.368 U/mg at substrate concentration 0.200 mM and incubation time 42 h and low LiP activity of 25.483 U/mg at substrate concentration 0.115 mM at 42 h (Table 2). The response surface curves shown in Fig. 8c represented that the LiP activity is regulated with the change of temperature and incubation time while the levels of pH and substrate concentration were kept unchanged. There was a peak in LiP activity recorded with the value of 55.947 U/mg at temperature 30 °C and incubation time 42 h. The lowest LiP activity of 25.483 U/mg was reported at temperature 37.5 °C and incubation time 42 h in the same condition as above (Table 2). Figure 8d described the response surface curves in which LiP activity is regulated in respect with substrate concentration and pH where the levels of temperature and incubation time kept in fix. In this condition, the highest LiP activity of 55.947 U/mg at substrate concentration 0.115 mM and pH 10.5 has again been recorded. Whereas, the lowest LiP activity of 25.483 U/mg was documented at substrate concentration 0.115 mM and pH 8.0 (Table 2). The response surface curves in Fig. 8e also showed a high LiP activity of 55.947 U/mg at temperature 30 °C and pH 10.5. Likewise, as in the condition described in Fig. 8d, lowest LiP activity of 25.483 U/mg has been measured at temperature 37.5 °C and pH 8.0 in the condition maintained in Fig. 8e. Figure 8f shows the response surface curves in which LiP activity have shown in respect with substrate concentration and temperature where the levels of incubation time and pH were fixed. This experimental condition also demonstrated the maximum activity of LiP with a value of 55.947 U/mg at temperature 30 °C and substrate concentration 0.115 mM but a low LiP activity of 25.483 U/mg at temperature 37.5 °C and substrate concentration 0.115 mM (Table 2). The residual plot analysis (Fig. 9) of LiP activity of the ligninolytic bacterial strain *Bacillus mycoides* represented a high degree of agreement in both the predicted vs. actual responses.

## Discussion

The isolation of bacteria and screening of their enzyme production ability are important and primary steps to establish their applicability in biotechnological industries. In the current study, a number of ligninolytic bacteria were isolated from decomposed leaf litter containing forest soils of Simlipal Biosphere Reserve and evaluated for their ligninolytic activity primarily using methylene blue dye as indicator and secondarily by culturing in broth medium containing alkali lignin. Similar study was undertaken by Jadhav et al. [49] for isolation of ligninolytic bacteria from different sources such as decomposing plant material, compost feed-stock, and decaying bark sample from rhizospheric soil. There are reports which showed that a diverse bacterial community can be abundantly found in top surface of forest soils as they are rich in decomposed plant litter materials [50, 51]. In the present study, 16 bacterial isolates were screened in LB agar medium containing methylene blue as an indicator for the determination of their ligninolytic activity. The bacterium SLB10 was found to show the highest decolourization zone with a percentage of 33.02 among all the screened bacterial strains. The present finding depicts the potential of highest lignin metabolism by SLB10 isolate and hence a possible higher LiP activity. Tian et al. [52], in their study, also screened some bacterial strains capable of metabolize lignin and lignin-related compounds for which they have used the methylene blue dye as an indicator in the bacterial growth medium for the screening purpose. Decolourizing effects of methylene blue, malachite green, and methyl orange catalyzed by the LiP enzyme from the fungus *Phanerocheate chrysosporium* was reported in the study of Alam et al. [53]. Their study revealed a maximum decolourization of 14% in case of methylene blue with compared to other two dyes which evidenced about the applicability of methylene blue effectively in the determination of dye decolourizing activity. The comparative decolourizing pattern as found in the present study and the investigation from Alam et al. illustrates that methylene blue can be used for determining the decolourization activity of both the bacterial and fungal LiP enzymes [53].

The bacterial isolates (SLB8, SLB9, and SLB10) were subsequently subjected to lignin peroxidase enzyme assay with a view to evaluate their comparative enzyme activity. From the results obtained from enzymatic assay of LiP, the highest activity has been noted for the isolate SLB10 with a value of 31.711 U/mg. LiP assay was performed using H_2_O_2_ as substrate and methylene blue as an indicator. The same procedure for the enzyme assay of LiP was followed by Vignali et al. [54] where the LiP activity of 9.5 U/mg was recorded for the bacteria *Rhodococcus jostii* which is approximately 3.34 times less compared to the LiP activity determined in the present study.

A bacterial growth pattern has also been analysed in the current investigation which showed a change in the size of the bacterial population over the time in the culture for the isolate SLB10. The highest growth or population size for the bacterium SLB10 has been recorded after the 24 h of incubation with a viable cell count log_10_ CFU/ml of 7.2833. Eastman et al. [55] has also studied the growth of *Paenibacillus polymixa* CR1 on lignin in presence of lignin in liquid culture with the terms of log_10_ CFU/ml per hour. The result from their study presented that the growth of the bacterium reached a maximum after 40 h of incubation showing viable cell count log_10_ CFU/ml of 7.2577 [56]. Therefore, in comparison with the result obtained from the study of Eastman et al., it can be stated that the strain SLB10 is an efficient strain capable of lignin biodegradation at a faster rate (maximum growth at 24 h of incubation) than that of bacterium *Paenibacillus polymixa* CR1 which took 40 h incubation time to reach maximum growth.

In order to determine the structure and chemical makeup of bacterial cell wall, gram staining method was presumed. The biochemical characterization was also carried out for the analysis of nutritional uptake and metabolic capabilities of bacteria using Bergey’s Manual of Systematic Bacteriology. The results obtained from gram staining showed that the isolate SLB10 is a rod-shaped and gram-positive bacterium. Different biochemical characterization of the bacterium SLB10 exhibited positive results in urease test, methyl red test, Voges-Proskauer test, and carbohydrate test and negative results in catalase test, oxidase test, citrate utilization test, and triple sugar iron test. The above results indicate that the bacterium SLB10 belongs to the genus *Bacillus*. Morphological and biochemical identification of some ligninolytic bacterial isolates from Kuthrel agro field of Bhilai-Durg region, Raipur has also been performed in the study by Naz [24]. In their work, the isolates were identified as the genus of *Bacillus* and *Streptomyces* with reference to Bergey’s Manual of Systematic Bacteriology. Those isolates were found to have the potential to tolerate high concentrations of lignin and production of ligninolytic enzymes.

Further, the 16S rRNA sequencing method was performed for the molecular identification of the bacterium SLB10 up to species level. Based on the sequencing and phylogenetic analysis showed that the bacterium SLB10 has the maximum homology with *Bacillus mycoides* and identified as *Bacillus mycoides*. In a similar type of study, Harith et al. [22] identified 8 ligninolytic bacteria and classified them as *Klebsiella* sp., *Enterobacter* sp., and *Bacillus cereus* on the basis of the partial sequence of 16S rRNA gene. In another study, Abd-Elsalam and El-Hanafy [19] performed partial sequence of 16S rRNA gene of some ligninolytic bacterial strains and identified two Bacillus strains such as *Bacillus subtilis* and *Bacillus* sp.. Therefore, it can be presumed that bacterial isolates belong to the genus *Bacillus* is common ligninolytic bacteria secreting LiP enzymes.

The chemical optimization of lignin peroxidase enzyme activity was conducted in presence of different inorganic nutrients like carbon, nitrogen, and metal ion sources. It was found from the current study that cellulose highly promotes the lignin peroxidase activity and maximizes the percentage of dye decolourization among all the carbon sources. Ramachandra et al. [56] in their study also reported an increased in lignin peroxidase activity in the presence of cellulose as carbon source where a higher consumption of hydrogen peroxidase has been noted during the catalytic reaction by the enzyme (Table 4). The effect of nitrogen source on LiP activity demonstrated the highest enzymatic turnover in case of yeast extract supplemented basal medium. Falade et al. [57] in their study reported that the composite effects of two nitrogen sources *viz.* yeast extract and ammonium sulphate enhanced LiP activity of *Bacillus* sp. MABINYA-1. Therefore, from this observation, it can be implied that yeast extract have significant impact of increasing LiP activity when applied alone or in combination with other nitrogen source. Studies on the effect of metal ion sources on LiP activity revealed that both the MnSO_4_ and KCl have significantly upregulated the LiP activity. There is a report that manganese in the form of MnCl_2_ as a metal ion source can increase the LiP activity in case of bacterium *Rhodococcus jostii* (Table 4) [54]. Therefore, the results obtained from the present study and the findings from Vignali et al. are in well agreement with the fact that manganese ion play an important role in enhancing the LiP activity.

**Table 4:** Comparison of lignin peroxidase activity in different bacterial strains

Bacteria	Substrate	Optimal physical parameters	Optimal chemical sources	Lignin peroxidase activity (U/mg)	References
*Rhodococcus jostii*	H_2_O_2_—0.1mM	pH—6.0 Temp—37 °C Time—24h	MnCl2—2 mM	9.5	[50]
*Streptomyces viridosporus*	2,4-dicholorophenol—1.0mM	Time—3 days	Cellulose	0.300	[52]
*Bacillus* sp. MABINYA-1	Kraft lignin	pH—5.0 Temp—30 °C Time—72 h	Glucose Yeast extract—0.1g/L Kraft lignin—0.1%w/v	17.50± 0.10	[53]
	Saw dust			47.14±0.41	
	Maize Stover			37.09±0.00	
	Wheat straw			21.65±0.35	
*Ensifer adhaerens* NWODO-2	Saw dust	pH—7.0 Temp—30°C Time—48 h	NM	37.50	[58]
	Wheat straw			5.25	
	Corn stover			3.76	
*Nonomuraea gerenzanensis*	2,6-Dimethyl phenol	pH—5.0 Temp—25°C	NaCl—1M DMSO—30% v/v Twin-80—1% v/v	1.98±0.08	[59]
	H_2_O_2_			2.84±0.17	
	ABTS			2.19±0.05	
	Catechol			2.81±0.13	
*Acinetobacter* sp.	n-Propanol	pH—2.0 temp—60 °C	NM	1.571	[60]
*Acinetobacter calcoacetius*	n-Propanol—100 mM	pH—1.0 temp—70°C	NM	0.045	[61]
*Deinococcus radiothilus*	2,4-Dichlorophenol	Time—3 days	NM	0.300	[62]
*Acinetobacter* sp. SW 30	n-Propanol—1.0 mM	pH—2.0 Temp—40 °C	NM	29.4	[12]
*Bacillus mycoides*	H_2_O_2_—0.115mM	pH—10.0 Temp—30°C Time—42h	Cellulose Yeast extract MnSO_4_	55.947	Present study

*NM* not mentioned

Further, in the present investigation, response surface methodology (RSM) was applied to optimize the culture conditions based on some important factors like pH, temperature, substrate (H_2_O_2_) concentration, and incubation time to get maximum LiP activity from *Bacillus mycoides*. In this study, the fit statistics calculation from the RSM indicates a low CV value which implied the designed experiment is highly reliable [63]. The *R*^2^ value obtained from the designed experiment for the optimization of *B. mycoides* LiP activity has been found as 0.9722 which is much closer towards 1.0. Doddapaneni et al. [58] and Haaland [59] suggested that closer the value of *R*^2^ to 1.0, the stronger the model and the better it predicts the response of tested enzyme activity according to designed conditions of ANOVA experiment. In the fit statistics of the response model of LiP activity, the value of adjusted *R*^2^ was less than the *R*^2^ value. Nooraziah and Tiagrajah [60] demonstrated that the adjusted *R*^2^ value may be smaller than the *R*^2^ when there are substantial terms in the model and the sample size is small. Some experimental factors might show to have significant emphasis in regulating enzymatic activity among all the interacting terms tested during RSM. Based on the *p* value calculation in coefficient table, the two experimental factors substrate concentration and temperature have the most effectiveness in maintaining LiP activity. Similarly, in the study of Paul et al. [26], highest xylanase activity from the isolated xylanolytic bacterium *Pseudomonas mohnii* was reported to be mostly influenced by the factors such as pH and its substrate corn cob xylan concentration.

In this study, optimization of culture condition using RSM showed that the enzyme LiP from *Bacillus mycoides* has the maximum activity of 55.947 U/mg at pH 10.5, temperature 30 °C, substrate concentration 0.115 mM, and incubation time 42 h. Though an optimum pH of 10.5 was determined in which highest LiP activity (55.947 U/mg) was observed yet at pH 8.0, an enhanced LiP activity of 37.155 U/mg has been recorded in two different experimental conditions where other three factors *viz.* temperature, substrate concentration, and incubation time differs. This observation suggests that an alkaline pH range between 8.0 and 10.5 may stimulate the increment in LiP activity of the strain *Bacillus mycoides*. Likewise, in a previous study by Falade et al. [61], LiP activity of 37.50 U/mg at optimum pH of 7.0 was recorded from the bacterium *Ensifer adhaerens* NWODO-2 (Table 4). From these observations, it can be concluded that maximum LiP activity is regulated under a broad range of alkaline pH condition. Other than alkaline pH, maximum LiP activity has also been recorded in three optimized experimental factors *viz.* substrate (H_2_O_2_) concentration of 0.115 mM, temperature at 30 °C, and incubation time of 42 h. Vignali et al. [54], in their study, have reported an almost similar H_2_O_2_ concentration (0.1 mM) as an optimized condition for the maximum LiP activity of 9.5 U/mg in *Rhodococcus jostii* (Table 4). Casciello et al. [62] have determined the LiP activity from the bacterium *Nonomuraea gerenzanensis* using the substrate H_2_O_2_ where LiP activity of 2.84 ± 0.17 U/mg was recorded. The optimized LiP activity (55.947 U/mg) of *B. mycoides* as calculated in the present study was found to be 19.7 fold greater than the LiP activity observed in case of *N. gerenzanensis* as reported by Cascielo et al. Apart from H_2_O_2_, there are several substrates like n-propanol, 2,4-dichloro phenol, 2,6-dimethyl phenol, ABTS, catechol, kraft lignin, maize stover, wheat straw, and corn stover which have reported to be used as substrate to estimate the LiP activity in various bacterial species [56, 64–66]. The optimum temperature (30 °C) and incubation time (42 h) for maximum LiP activity as obtained in the present study is also in well agreement with the investigation conducted by Falade et al. [61], where maximum Lip activity was obtained at temperature 30 °C and incubation time 48 h (Table 4). There are only two reports available till date on optimization study related to total bacterial LiP activity using RSM. Saha [67] demonstrated the optimization of LiP activity of the bacterium *Paenibacillus mucilaginosus* S-4, and the maximum total enzyme activity was recorded as 22.825 U/ml. Another work was conducted by Rizk et al. [68], where optimization of LiP production and its enzymatic activity have been demonstrated in aquatic bacterium, *Alcaligenes aquatilis*. For optimizing the enzyme activity and its production, Plackett-Burman design and Central composite design have been used respectively in this study. The results of the LiP activity in this study showed that the most optimum incubation time and pH were 24 h and 6.0, respectively, in which the highest total LiP activity of 3.35 U/ml has been noted [59]. However, in the present work, the optimization of LiP activity using Box-Behnken design from the bacterium *Bacillus mycoides* isolated from forest soil sample is a new study as no such investigation has been undertaken previously. It is also noteworthy that in this present work, the highest LiP activity of 55.947 U/mg was obtained from *B. mycoides* calculated in terms of specific activity of the enzyme. This specific LiP activity from *B. mycoides* is even greater than the LiP activity (47.14 ± 0.41 U/mg) calculated using saw dust as substrate from the bacterium Bacillus sp. MABINYA-1 (Table 4) [57], and this is still highest among all the bacterial LiP activity reported till date. Therefore, it can be stated from these comparative results that the bacterial strain isolated from forest soil samples of SBR is an efficient LiP producing candidate which can serve in potent lignin biodegradation.

## Conclusion

Current study depicted that the soils collected from the forests of Simlipal Biosphere Reserve (SBR) of Odisha, India, are a rich source of lignocellulolytic bacteria with high lignin peroxidase activity. The present study revealed the potential of the strain SLB10 for high lignin peroxidase enzyme production. The ligninolytic strain SLB10 obtained from the soil of SBR was identified as *Bacillus mycoides* through morphological, biochemical characterization, and 16S rRNA sequencing. The lignin peroxidase enzyme activity of this bacterium was 31.711 U/mg under unoptimized condition. The optimization through RSM the ligninolytic activity resulted in a higher lignin peroxidase activity (55.947 U/mg) of the ligninolytic bacterium *B. mycoides*. The enzyme production showed approximately 1.76 folds enhance over the un-optimized condition. Individually, the presence of cellulose, yeast extract, and MnSO_4_ in the culture medium of *B. mycoides* promotes both the LiP activity and dye decolourization. The present study thus highlights the potential of the newly isolated bacterium *B. mycoides* from SBR for possible use for large-scale enzyme production through adoption of appropriate technology. The enzyme production from this bacterium can be used for industrial purpose including bioethanol production, textile industries, toxicity reduction in pulp and paper mill effluents, cosmetology, and dermatology through its novel mechanism. However, additional work related to protein characterizations and pilot scale production of the LiP enzyme from this bacterium need to be studied.

## Data Availability

All data generated or analyzed during this study are included in this article.

## Abbreviations

SBR: Simlipal Biosphere Reserve
SLB: Simlipal ligninolytic bacteria
RSM: Response surface methodology
KCl: Potassium chloride
MgSO_4_: Magnesium sulphate
MnSO_4_: Manganese sulphate
ZnSO_4_: Zinc sulphate
CuSO_4_: Copper sulphate
FeSO_4_: Ferrous sulphate
NaCl: Sodium chloride
CaCl_2_: Calcium chloride
CoCl_2_: Cobalt chloride
NaNO_3_: Sodium nitrate
NH_4_NO_3_: Ammonium nitrate
H_2_SO_4_: Sulphuric acid
MSM-L: Minimal salt medium-lignin
LCB: Lignocellulosic biomass
